# A Cheaper Alternative to Conventional Mid-urethral Sling Surgery Can Also Have Comparable Obstetric Outcome in the Long Term: A Case Report

**DOI:** 10.7759/cureus.73109

**Published:** 2024-11-06

**Authors:** Abhijit Mondal, Dibyendu Roy, Komal Singh

**Affiliations:** 1 Obstetrics and Gynecology, Tata Main Hospital, Dhanbad, IND; 2 Obstetrics and Gynecology, Calcutta National Medical College, Kolkata, IND; 3 Obstetrics and Gynecology, Tata Central Hospital, Dhanbad, IND

**Keywords:** alternate approach, follow-up, obstetric outcome, spontaneous vaginal delivery, sui

## Abstract

Stress urinary incontinence (SUI) is a common disorder in females, which significantly affects the quality of life in females. There are many consensus that describe the safety of childbearing after surgery for SUI, but still, a large proportion of surgeons worldwide recommend that women should wait to complete childbearing before pursuing surgical treatment for SUI. There is also some opinion that if patients conceive after surgical treatment for SUI, women should be delivered by cesarean section. Trans obturator tape (TOT) procedure is the most commonly performed mid-urethral sling (MUS) surgery for the treatment of SUI. We are reporting a case where a P2+0 woman underwent a TOT procedure where we used a cheaper alternative to a conventional TOT kit. She conceived and underwent spontaneous vaginal delivery after the procedure. She remained continent in the follow-up even after one year of vaginal delivery.

## Introduction

Stress urinary incontinence (SUI) is a common condition reported to affect 25% to 57% of women [1]. The peak age at onset is 45 to 49 years. With a reported prevalence of 10.8% to 14%, SUI occurs less frequently in women younger than 40 years than in older women [2]. Pregnancy and childbirth are the biggest risk factors of SUI. Vaginal birth is known to have a major impact on the pelvic floor, weakening bladder neck support. According to many surgeons worldwide, it is necessary to delay surgical intervention for SUI till childbearing is completed. SUI is diagnosed from clinical assessment and urodynamic studies (UDS) [3]. The mid-urethral sling (MUS) procedure is considered to be the "gold standard" treatment of SUI [4]. Elective cesarean section is considered to be the method of delivery by many obstetricians worldwide in women who conceived after surgery for SUI. In this case, we surgically managed a patient with SUI where we used an indigenous TOT needle and hernia mesh to reduce the cost of surgery, and later on, she conceived and delivered a full-term baby by vaginal delivery without any adverse effect on the primary surgical procedure.

## Case presentation

A 30-year-old P2+0 lady from low socio-economic status with a previous history of two home vaginal delivery attended our outdoor clinic. The local 'Dai' performed a vaginal delivery. With a history of urinary incontinence while straining for the past two years, she visited our hospital's gynecology outpatient department (OPD). The constant leakage was uncomfortable and had a detrimental effect on her life. Despite seeking medical help from different hospitals, her symptoms continued to increase gradually. She had no past instances of burning or increased frequency of urination. Her BMI was 31, and her urine analysis results were normal. On per speculum examination urine leakage on straining was present. Cotton swab/ Q-tip test confirmed urethral hypermobility indicative of second-degree SUI. Initial conservative management, including weight reduction and Kegel exercises, was unsuccessful after three months. Due to the patient's desire for future pregnancy, surgical intervention for SUI was postponed until after childbirth. However, the patient's quality of life was significantly impacted, leading to a strong desire for immediate surgery. 

**Figure 1:**
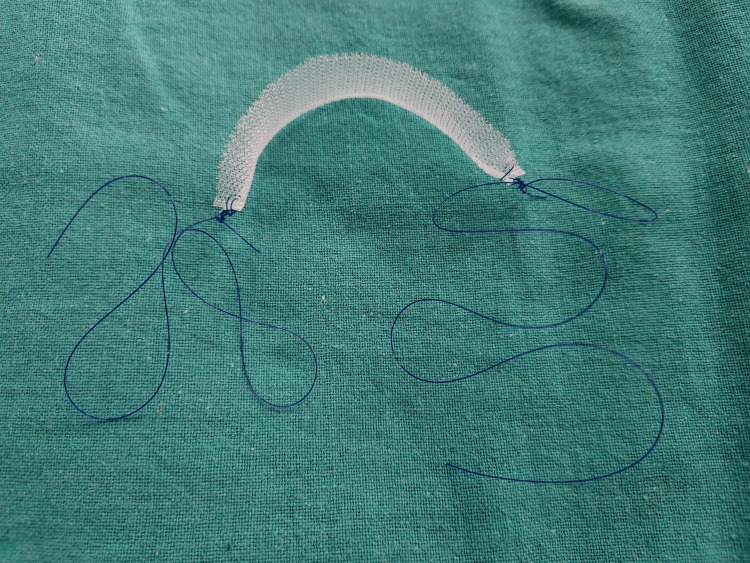
Hernia mesh and prolene suture used in place of conventional TOT system We used 11cm prolene hernia mesh along with prolene suture in place of a conventional TOT system to reduce the cost of the procedure. TOT - trans obturator tape

**Figure 2:**
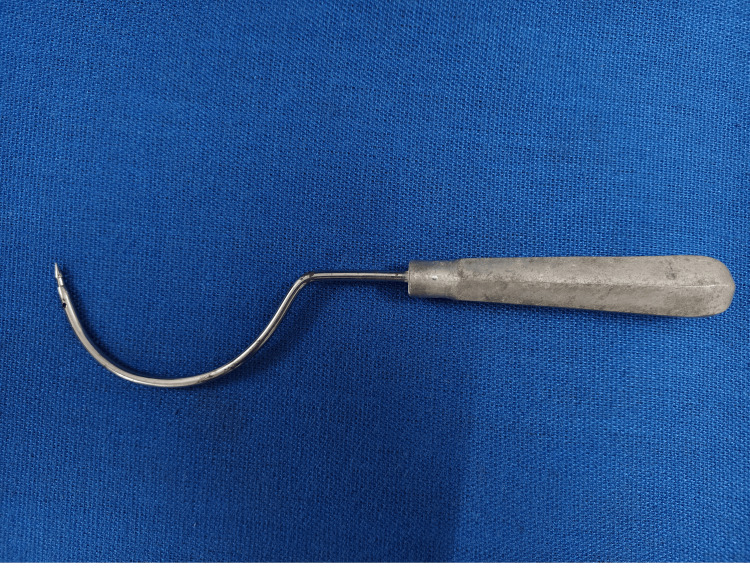
Indigenous TOT needle TOT - trans obturator tape

The patient was counseled on the potential need for a cesarean section and the risk of SUI recurrence after delivery. Considering the patient's low income, a cost-effective surgical approach was employed. Instead of the typical trans obturator tape (TOT) system, prolene mesh, commonly used for hernia repair, and prolene suture with an indigenous needle were utilized (Figures 1, 2), reducing the procedure cost by approximately ten times. Post-operatively, the patient recovered well.

**Figure 3:**
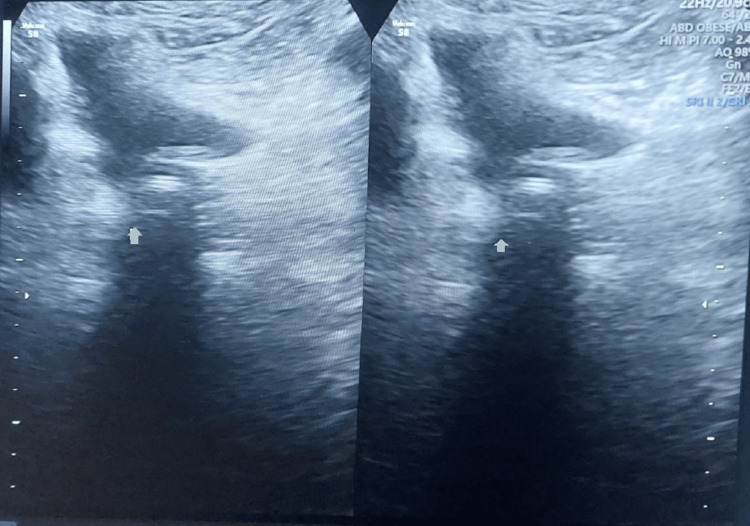
Post-delivery transvaginal sonography showing mesh in proper position We have done a post-delivery transvaginal sonography, which shows mesh is in the proper place, visible as an echogenic linear shadow

One year after surgery, the patient became pregnant and received regular antenatal care. With no medical or obstetrical complications, she was allowed to wait for spontaneous labor. She delivered a healthy baby weighing 3 kg. Post-delivery, the patient underwent regular follow-up to monitor for SUI recurrence. She remained continent one year after childbirth. A transvaginal ultrasound performed one year post-delivery confirmed the mesh was in the proper position (Figure 3).

## Discussion

International Continence Society defined SUI as "the complaint of involuntary leakage on effort or exertion, or on sneezing or coughing". The sign observed by the physician to verify or quantify the symptom of SUI is described as "the observation of involuntary leakage from the urethra, synchronous with exertion/effort, or on sneezing or coughing".

It is reasonable to consider initiating treatment for SUI in any patient with significant symptoms of SUI without performing urodynamic studies [5]. Vaginal delivery is a known risk factor for stress urinary incontinence as it causes damage to the structure and function of the pelvic floor.

In comparison to vaginal delivery, cesarean delivery is associated with a reduced rate of urinary incontinence and pelvic organ prolapse [6]. Evidence suggests that the prevalence of both urge and stress incontinence increases proportionately with rising BMI [7]. In managing urinary incontinence, it is essential to identify and address potential reversible contributors. Research indicates that a balanced diet, regular bowel movements, weight loss, and physical exercise can be beneficial in conservatively managing stress urinary incontinence. In the case of our patient, who had a BMI of 31, we advised her to reduce her weight and engage in Kegel exercises to alleviate her symptoms.

Despite conservative management, our patient's symptoms persisted, prompting us to consider surgical intervention. According to the Cochrane database system review, mid-urethral sling surgeries, such as the trans obturator tape (TOT) procedure, are highly effective in managing stress urinary incontinence, regardless of the delivery route [8]. Given our patient's history of two previous vaginal deliveries and the development of stress urinary incontinence one year after her last delivery, we opted for the TOT procedure as the best available surgical option to address her symptoms. She was counseled properly regarding the procedure and complications associated with the procedure, like urge symptoms, dyspareunia, perineal pain, and vaginal erosions.

Conventionally, TOT is done by using a pre-packed TOT kit, which has a TOT needle and mesh, which usually costs around 26,000 INR to 32,000 INR to the patient. In our case, instead, we used a TOT needle and normal hernia repair mesh, which cost the patient around 2600 INR. This customization of using hernia mesh along with prolene suture material helped to reduce the cost of the procedure by 10 times. As the patient belongs to a low socio-economic status, this cost-effectiveness proved to be very helpful for the patient. Our patient conceived one year after surgery. She was in regular follow-up in the antenatal period in our OPD. Our patient had no significant medical comorbidities, and she also had no other obstetrical indication for the cesarean section. Eventually, she delivered a healthy baby at term vaginally, weighing 3 kg. On subsequent follow-up, the patient remained continent even one year after delivery, and the mesh position was confirmed by transvaginal sonography.

Previously, cesarean section was the preferred route of delivery for patients who underwent sling surgeries due to the fear of SUI recurrence.

But according to Huser et al., the risk of SUI recurrence is not significantly different after a vaginal or a cesarean delivery [9]. In women successfully treated with a MUS, pregnancy care and delivery mode, therefore, need to be individualized according to factors other than the risk of recurrence.

## Conclusions

SUI reaches a peak at around the fifth decade of life in females, although women of younger age can also develop this condition. SUI significantly affects women's quality of life. Management can be initiated in patients with significant symptoms without performing urodynamic studies. MUS surgery is a recommended procedure for SUI. Patients with MUS surgery can undergo vaginal delivery without complications, provided done cautiously. The cost of the surgery can be reduced by 10 times using the prolene mesh and indigenous TOT needle. This customization also gives comparable long-term obstetric outcomes to other TOT kits available in the market, which can be beneficial for patients of low socio-economic status.
